# Amino-Acid-Derived Toxins and Pyrazines in Chocolates and Cocoa-Free Chocolate Surrogates

**DOI:** 10.3390/molecules31071148

**Published:** 2026-03-31

**Authors:** Alexandre Dusart, Lucie Villé, Thomas Vantsiotis, Sonia Collin

**Affiliations:** Unité de Brasserie et des Industries Alimentaires, Louvain Institute of Biomolecular Science and Technology (LIBST), Faculté des Bioingénieurs, Université catholique de Louvain, 1348 Louvain-la-Neuve, Belgium

**Keywords:** α,β-unsaturated carbonyls, cocoa, amino acids, Strecker degradation, pyrazines, chocolate surrogates, genotoxicity

## Abstract

This study investigated the influence of cocoa-bean amino acid profile on the formation of α,β-unsaturated carbonyls. Some of these heat-derived compounds generated primarily during roasting (often through Strecker degradation and aldol condensation) are of toxicological concern (e.g., the aneugenic 2-phenylcrotonaldehyde and the genotoxic furan-2(5*H*)-one). Here, their levels were compared by SAFE-GC/MS in cocoa of different origins, before and after roasting. Free amino acid profiles were determined by HPLC-MS/MS. All the investigated roasted beans showed similar total free amino acid contents (10.5–15.0 g·kg^−1^) and profiles, while α,β-unsaturated aldehyde levels differed markedly between samples, and reached >200 µg·kg^−1^ after roasting, indicating that amino acid availability alone does not govern their formation. Analysis of a commercial cocoa-free chocolate surrogate, however, revealed much lower amounts of both free amino acids (total 1.6 g·kg^−1^) and amino-acid-derived α,β-unsaturated aldehydes (total 11 µg·kg^−1^) than in chocolate, together with only traces of pyrazines (total 72 µg·kg^−1^). In contrast, furan-2(5*H*)-one was found at similar levels in chocolate and the cocoa-free product (56–79 µg·kg^−1^), confirming a completely different pathway where amino acids are not key reagents in its synthesis.

## 1. Introduction

**Figure 1:**
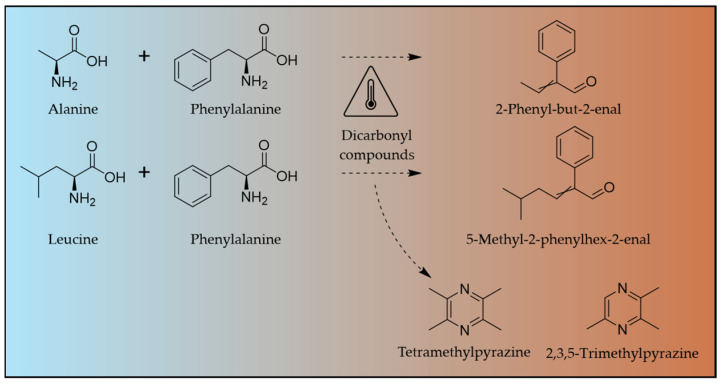
Typical heat-related compounds produced by amino acid thermal degradation.

Cocoa bean variety, pedoclimatic conditions, and post-harvest processing are key determinants of chocolate quality. During roasting (typically 30 min at 110–150 °C), heat-induced reactions generate characteristic flavour compounds such as 2- and 3-methylbutanal (malty, cocoa-like), phenylacetaldehyde (floral), and various pyrazines, mainly through Strecker degradation of amino acids [1,2]. Yet these same pathways can also lead to the formation of undesirable compounds [3]. Aldol condensation between two Strecker aldehydes, followed by dehydration, can yield α,β-unsaturated aldehydes [4,5] (Figure 1). Some of them are of toxicological concern, such as aneugenic 2-phenylcrotonaldehyde, whereas others remain under evaluation [6]. Also identified in chocolate is the heat-derived genotoxic compound α,β-unsaturated carbonyl furan-2(5*H*)-one [7]. Unlike Strecker-derived α,β-unsaturated aldehydes whose formation depends on amino acids, the formation pathway of furan-2(5*H*)-one has not yet been fully elucidated. Proposed mechanisms include oxidation of furfuryl alcohol followed by decarboxylation steps, without direct involvement of amino acids [7]. Used as a flavouring substance until 2019, furan-2(5*H*)-one is no longer authorised to be used as such in foodstuffs, but its broad natural occurrence is of concern for public health [7,8].

Recent studies have shown that cocoa varieties differ substantially in their propensity to generate these compounds [7]. Brazilian *Forastero* beans, for example, appear better suited for producing high amounts of pyrazines, while Haitian *Criollo* beans generate more Strecker aldehydes and derived toxins. These differences may reflect the free amino acid composition, since free amino acids are the primary precursors of both desirable and undesirable heat-generated compounds. Fine cocoa varieties such as *Criollo* have been shown to contain over 10% more amino acids than bulk varieties (e.g., *Forastero*), contributing to their richer aromatic profile [9]. Yet the free amino acid content alone is not usually fully predictive of aroma formation [9,10]. In fact, beyond genetic factors, post-harvest processes (e.g., fermentation and drying) influence proteolysis and hence free amino acid levels [5]. Roasting and conching conditions also affect the Maillard and Strecker reactions and the release of newly formed volatiles through evaporation. More generally, pedoclimatic conditions can also influence the composition of cocoa beans.

Alongside traditional cocoa and cocoa products, the food industry is actively developing, in response to environmental, economic, and supply chain pressures, cocoa-free chocolate surrogates as more sustainable alternatives to conventional chocolate [11]. These products aim to reproduce the sensory attributes of chocolate, often with combinations of cereals, vegetables, or other plant-based ingredients [12,13,14,15]. Several patents are already published or in pending status [16,17]. Yet, to date, the chemical compositions of such substitutes, and particularly their aromatic profiles, remain largely unexplored. No detailed investigations have yet assessed whether they can generate α,β-unsaturated carbonyls during processing, nor whether their free amino acid content supports the development of typical chocolate-like aromas.

The aim of this work was first to investigate the amino acid profiles, determined by high-performance liquid chromatography coupled with tandem mass spectrometry (HPLC-MS/MS), of different cocoa beans in relation to their propensity to generate α,β-unsaturated carbonyls and pyrazines through roasting (determined by gas chromatography coupled to mass spectrometry (GC-MS) after solvent-assisted flavour evaporation (SAFE) extraction). Beans were deliberately selected to provide a contrasted set of samples differing in origins, genetic background, pedoclimatic conditions, and post-harvest processing. Secondly, the same techniques were used to compare a recently commercialised cocoa-free chocolate surrogate made from sunflower and grape seeds with two bean-to-bar chocolates, one made from Malagasy and one from Brazilian cocoa beans.

## 2. Results

### 2.1. Cocoa Bean Free Amino Acid Profiles Determined by HPLC-MS/MS

The six roasted cocoa bean samples analysed showed very similar free amino acid profiles and total free amino acid contents (Table 1). In all cases, leucine, isoleucine, and phenylalanine (precursors of 3-methylbutanal, 2-methylbutanal, and phenylacetaldehyde, respectively) emerged as the major structures, accounting for 47–58% of total free amino acids, which themselves represent 1.0–1.5% of cocoa bean weight.

### 2.2. Cocoa Bean α,β-Unsaturated Carbonyls (Before and After Roasting) Determined by SAFE-GC-MS

**Figure 2:**
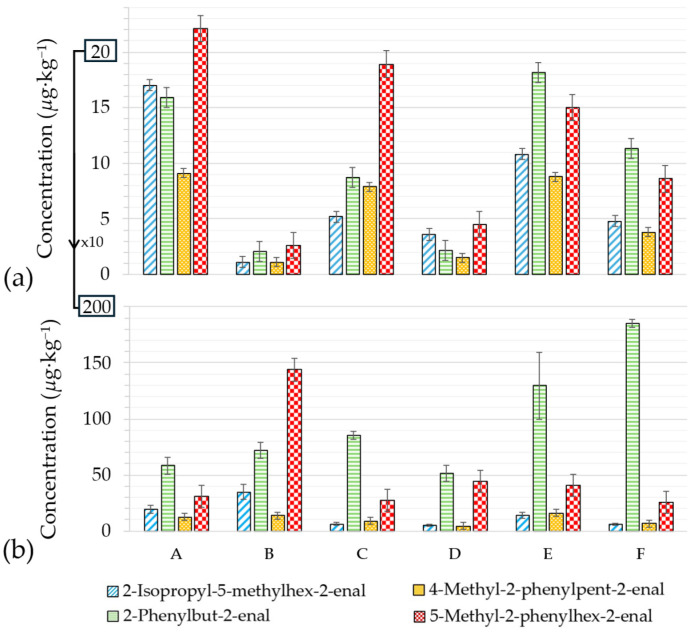
Levels of α,β-unsaturated aldehydes (µg·kg^−1^) in cocoa beans (**a**) before and (**b**) after roasting for 30 min at 140 °C (A: Madagascar, B: Brazil, C: Venezuela, D: Mexico, E: Cuba, F: Cameroon).

Among the suspected genotoxic α,β-unsaturated aldehydes, 2-isopropyl-5-methylhex-2-enal, 2-phenylbut-2-enal, 4-methyl-2-phenylpent-2-enal, and 5-methyl-2-phenylhex-2-enal were detectable already in all unroasted cocoa beans (up to 22 µg·kg^−1^ of 5-methyl-2-phenylhex-2-enal in sample A) (Figure 2a). Their occurrence is likely due to the temperatures reached during fermentation (up to 50 °C) and to subsequent sun-drying processes [7].

Big differences appeared between samples, with much lower levels in Brazilian *Forastero* (B) and Mexican *Criollo* (D) (less than 5 µg·kg^−1^ of each). On the other hand, *Criollo* beans from Madagascar (A), *Chuao* beans from Venezuela (C), and *Trinitario* beans from Cuba (E) appeared more prone to generating these compounds (5–22 µg·kg^−1^).

After roasting, levels of these suspected genotoxic compounds increased in all samples (Figure 2b; scale ten times larger than Figure 2a). This rise confirms the key role of this step in their formation. 2-Phenylbut-2-enal and 5-methyl-2-phenylhex-2-enal showed the most substantial rises (up to 16- and 55-fold, respectively), while 2-isopropyl-5-methylhex-2-enal and 4-methyl-2-phenylpent-2-enal barely increased on average. The increase was greater in samples B (×38), D (×9), and F (×8), despite similar amino acid profiles.

**Figure 3:**
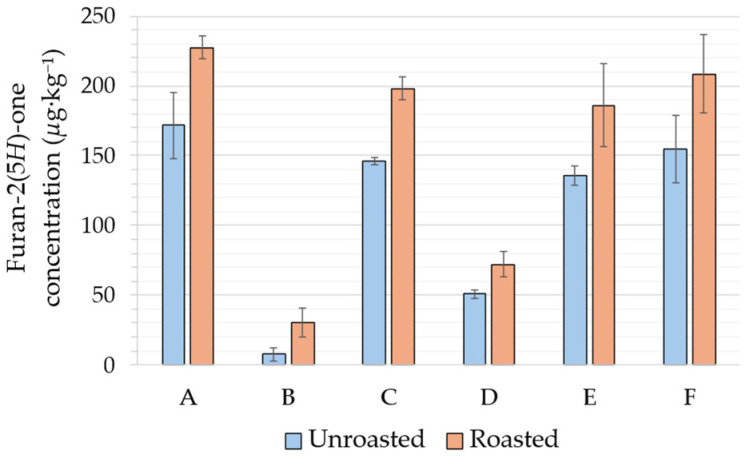
Furan-2(5*H*)-one level in cocoa beans before and after 30 min roasting at 140 °C (A: Madagascar, B: Brazil, C: Venezuela, D: Mexico, E: Cuba, F: Cameroon).

Interestingly, genotoxic furan-2(5*H*)-one was likewise found at lower levels in B and D (yet up to 51 µg·kg^−1^ in D) than in the other samples (Figure 3; levels close to 150 µg·kg^−1^ in A, C, E, and F). Fortunately, its increase through roasting was much more limited (×1.3–1.4 in A, C, D, E, F; ×3 in B), so levels remained below 228 µg·kg^−1^ in all samples.

### 2.3. Free Amino Acid Levels in Chocolate and Cocoa-Free Chocolate Surrogates

Free amino acid levels were further determined in the bean-to-bar chocolate (76.2% cocoa, 25% sugar) produced from the roasted beans from A (20 h dry conching, 6 h wet conching). As depicted in Table 2, leucine/isoleucine and phenylalanine remained the major amino acids after roasting and conching (here up to 8625 and 4463 mg·kg^−1^, respectively). A marked difference was observed between this chocolate and the cocoa-free chocolate surrogate, in terms of both total free amino acid content, which was substantially lower in the latter, and profiles of individual free amino acids (Table 2). It was expected that the formation of Strecker aldehydes derived from these amino acids should also be affected, in terms of both overall levels (expected to be lower) and compositional profiles. It was likewise anticipated that levels of α,β-unsaturated carbonyl compounds should be lower in the surrogate.

### 2.4. α,β-Unsaturated Carbonyls in Chocolate and Cocoa-Free Chocolate Surrogate

**Figure 4:**
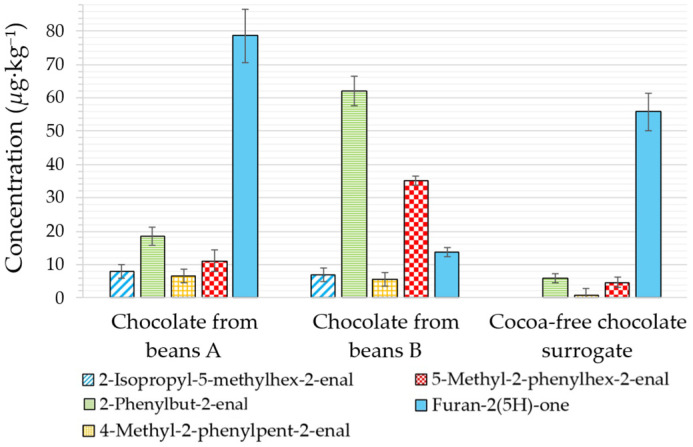
Levels of four α,β-unsaturated aldehydes and furan-2(5*H*)-one in chocolates from A and B cocoa beans, and in the cocoa-free chocolate surrogate.

The α,β-unsaturated aldehyde content, in line with reduced free amino acid levels, was indeed significantly lower in the surrogate than in chocolate (two samples here investigated—from beans A and beans B). Compared to the roasted beans, chocolates contained less α,β-unsaturated aldehydes (19 and 62 µg·kg^−1^ of 2-phenylbut-2-enal in chocolate A and B, respectively), due to dilution and evaporation during conching (Figure 4) [7]. This was also the case of the cocoa-free chocolate surrogate, albeit with much lower levels (in total, <11 µg·kg^−1^), as expected based on its amino acid content.

On the other hand, the α,β-unsaturated lactone furan-2(5*H*)-one was detected in the surrogate at levels typical of those found in chocolate (56 µg·kg^−1^ vs. 14–79 µg·kg^−1^, Figure 4). This confirms that amino acids are not its key precursors. In commercial sweet snacks, levels reach up to 4000 µg·kg^−1^; chocolate is therefore less worrying [7]. Nonetheless, a daily consumption of only 3 g of chocolate per person represents the maximal exposure that remains below the threshold of toxicological concern for genotoxic substances (assuming an average furan-2(5*H*)-one level of 50 µg·kg^−1^) [18].

### 2.5. Key Chocolate Flavours in Chocolate and Cocoa-Free Chocolate Quantified by SAFE-GC-MS

**Figure 5:**
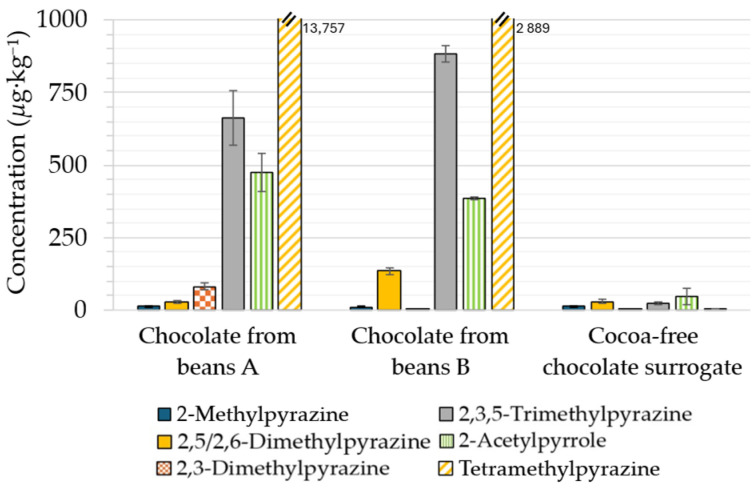
Levels (µg·kg^−1^) of nitrogen heterocycles in chocolates and in the cocoa-free chocolate surrogate.

Logically, many nitrogen heterocycles (Figure 5) were found above their sensory thresholds in both chocolates (e.g., 662–884 µg·kg^−1^ trimethylpyrazine (threshold: 290 µg·kg^−1^ [19]), 2900–13,800 µg·kg^−1^ tetramethylpyrazine (threshold: 5000 µg·kg^−1^ [20])). In contrast, only traces (<50 µg·kg^−1^ each) were found in the cocoa-free surrogate sample. These results are consistent with the occurrence of so few Strecker degradation reagents.

**Figure 6:**
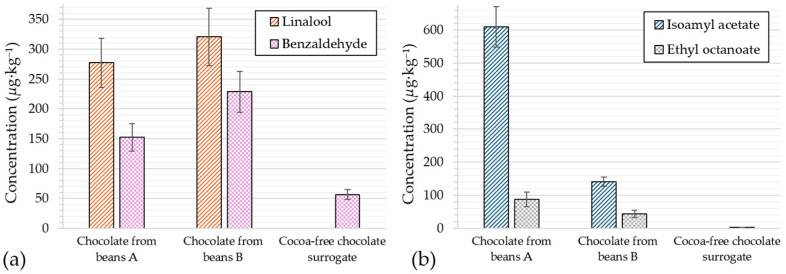
Levels of (**a**) varietal and (**b**) fermentation-derived flavours in chocolates and the cocoa-free sample.

The SAFE extracts were also analysed by GC-MS to quantify two varietal aromas, linalool and benzaldehyde, and two fermentation-derived products, isoamyl acetate and ethyl octanoate [21]. As shown in Figure 6a, coriander-like linalool (sensory threshold: 1.5 µg·kg^−1^ [22]) was completely absent from the cocoa-free sample, while levels of 277–320 µg·kg^−1^ were found in chocolates A and B. In contrast, almond-like benzaldehyde (threshold: 100 µg·kg^−1^ [20]) was found at 56 µg·kg^−1^, still about three times lower than the levels found in chocolates from A and B beans. Only negligible amounts (<3 µg·kg^−1^) of fermentation-derived products were detected in the cocoa-free chocolate surrogate. This provides further evidence that its volatile profile differs from that of chocolates.

As vanillin was not added in the bean-to-bar chocolate productions, levels below 1 µg·kg^−1^ were found in both chocolates. In contrast, 5440 µg·kg^−1^ was found in the cocoa-free chocolate surrogate, explaining its strong vanilla aroma. In addition, the relatively milkier notes from the cocoa-free chocolate surrogate could be due to the occurrence of 681 µg·kg^−1^ dimethylsulfone coming from the milk powder (only <1 µg·kg^−1^ in chocolate from A beans).

## 3. Discussion

In the present work, α,β-unsaturated aldehyde levels were examined across cocoa beans, chocolates, and a cocoa-free surrogate, in relation to the availability of free amino acids. The results demonstrate a partial dependence on amino acid availability. The cocoa-free surrogate, very poor in free amino acids, showed markedly lower amounts of both α,β-unsaturated aldehydes and typical chocolate aromas (e.g., pyrazines). Yet the inability to predict α,β-unsaturated aldehyde contents in chocolates from different bean origins (all with similar free amino acid profiles) indicates that amino acid availability alone does not govern their formation in cocoa.

In contrast, furan-2(5*H*)-one formation appeared independent of the availability of free amino acids. Similar levels were detected in both chocolates and in the cocoa-free surrogate, despite the latter’s low amino acid pool. This finding provides experimental support for the existence of amino-acid-independent formation pathways. Based on the present results and previous mechanistic proposals, furan-2(5*H*)-one formation is likely linked to carbohydrate-derived furanic intermediates, such as furfuryl alcohol, undergoing oxidation and subsequent decarboxylation reactions during thermal processing. While the present study does not allow direct identification of these pathways, the persistence of furan-2(5*H*)-one in the cocoa-free surrogate investigated here supports this hypothesis.

This study supports the development of processing strategies aimed at mitigating α,β-unsaturated carbonyls of concern in both chocolate and chocolate alternatives. Future research should investigate a broader range of cocoa-free chocolate surrogates (several will probably soon be commercially available).

## 4. Materials and Methods

### 4.1. Samples

Dried fermented cocoa bean samples were obtained from “Le Cercle du Cacao” (Evere, Belgium). Table 3 details the origin and the variety of the 6 bean samples.

Bean-to-bar chocolates were produced from either cocoa bean sample A or B, following the procedure described in [7]. Cocoa beans were roasted at 140 °C for 30 min. Afterwards, bean shells were discarded, and the remaining cocoa nibs were ground for 40 min with a robot-coupe (R8VVE model from Robot Coupe SNC, Vincennes, France). After 35 min of grinding, powdered sugar equivalent to one-third of the cocoa nib mass was added to the cocoa mass so as to obtain 25% sugar. Dry conching (Concher from Spectra Company, New York, NY, USA) took place for 20 h, with the cocoa mass being heated to 60 °C during the first hour. The conching lid was kept open during the first 2 h of conching (evaporation of residual acetic acid). The cocoa mass was at 50 °C at the end of dry conching. Wet conching started with adding cocoa butter equivalent to 7% of the cocoa mass (taking into account the mass removed for sample analysis). Wet conching continued for 6 h until a particle size of 18 μm was obtained (verified using an OTMT Electronic Micrometre from Otelo, Saint-Ouen-l’Aumône, France). The chocolate was then filtered through a metal sieve (mesh size: 500 µm) to avoid residual stone residue from the concher.

The cocoa-free chocolate surrogate “ChoViva” was purchased from ABTEY Chocolaterie (Heimsbrunn, France) in April 2025. The ingredients were the following: sugar, vegetable fat (palm and shea), skimmed milk powder, sunflower seed flour, grapeseed flour, emulsifier, and natural flavour.

### 4.2. Chemicals

2,3,5-Trimethylpyrazine, 2,3-dimethylpyrazine, 2,5-dimethylpyrazine, 2,6-dimethylpyrazine, 2-acetylpyrrole, 2-acetylthiophene, 4-methyl-2-phenylpent-2-enal, 5-methyl-2-phenylhex-2-enal, 2-methylpyrazine, *n*-decane, and tetramethylpyrazine were purchased from Merck (Overijse, Belgium). Furan-2(5*H*)-one was purchased from Fisher Scientific (Brussels, Belgium). Dichloromethane (>99.8%), ethanol absolute (99%), and diethyl ether (>99%) were purchased from VWR International (Leuven, Belgium). Anhydrous sodium sulfate was purchased from Merck (Overijse, Belgium).

Alanine, 3-aminoisobutyric acid, arginine, asparagine, aspartic acid, glutamic acid, glutamine, glycine, histidine, isoleucine, leucine, lysine, methionine, phenylalanine, proline, serine, threonine, tryptophan, tyrosine, and valine were purchased from Merck (Overijse, Belgium).

### 4.3. Free-Amino-Acid Analysis

#### 4.3.1. Isolation

Fifty grams of roasted cocoa beans (30 min at 140 °C) was manually dehulled and finely ground. Two aliquots (20 g) of the homogenised powder were collected for duplicate analysis. DL-3-Aminoisobutyric acid (20 mg) was added as an internal standard (IS). Samples were defatted by liquid–liquid extraction with diethyl ether (3 × 50 mL; 15 min stirring followed by 10 min centrifugation at 2700× *g*) [23]. The defatted cocoa powder was air-dried (~1 min) before amino acid extraction. One gram was extracted three times with ultrapure water (10, 5, and 5 mL), vortexed, and centrifuged (10 min at 3900× *g*); supernatants were pooled [24]. Proteins were precipitated by mixing 400 µL of extract with 1600 µL of acetonitrile, followed by vortexing and centrifugation (2 min at 15,000 rpm). The supernatant was filtered (0.2 µm) and stored at −80 °C until analysis.

#### 4.3.2. HPLC Analysis

Free amino acids were analysed using a UHPLC Vanquish Horizon system (Thermo Fisher Scientific, Waltham, MA, USA) coupled to a TSQ Quantis Plus Mass Spectrometer. Chromatographic separation of free amino acids was performed on a SeQuant^®^ Zic-HILICTM (50 × 2.1 mm i.d., 3.5 µm, 100 Å) from Supelco (Bellefonte, PA, USA) by using a gradient elution of acetonitrile (A) and water (B) at a flow rate of 1 mL/min at 30 °C. The gradient elution started with 80% A, was held for 2 min, and then linearly decreased to 0% in 1 min and was held for 3 min. Then, it was increased to the initial conditions (80% A) in 1 min and held for 3 min. Total chromatographic run time was 10 min. The injection volume was 1 μL.

#### 4.3.3. MS/MS and Quantification

MS source was used in the positive ionisation mode (ESI+). The electrospray source had the following settings: vaporizer temperature, 400 °C; capillary voltage, 3.5 kV; sheath gas flow, 8 L/min; auxiliary gas flow, 14.3 L/min; sweep gas flow, 2.7 L/min. Amino acids were identified by multiple reaction monitoring (MRM) using the parameters given in Table S1. External calibration of amino acids was performed. Three separate amino acid standard groups were created according to expected concentration ranges in the extracts (Table S2). Amino acids were prepared in pure water, except for tyrosine, which was solubilized in acidic water (pH 1, using HCl). The following equation was used for quantification, considering identical recovery factors between the analytes and the internal standard (IS): amino acid concentration (in mg·kg^−1^) = IS concentration (in mg·kg^−1^) × (amino acid area/IS area) × (IS response coefficient/amino acid response coefficient). The latter factor was determined by external calibration when analytical standards were available.

### 4.4. Volatile Analysis

#### 4.4.1. Isolation by Solvent-Assisted Flavour Evaporation (SAFE)

Volatiles were isolated from cocoa beans using solvent-assisted flavour evaporation (SAFE) as described previously [7,25]. All analyses were performed in duplicate. Finely ground cocoa samples (30 g) were spiked with 150 μL of 2-acetylthiophene solution (8000 µg·L^−1^) as an internal standard (concentration in sample: 40 μg·kg^−1^). Samples were extracted twice with bidistilled dichloromethane (2 × 50 mL) for 20 min, and the combined organic phases were filtered through glass fibre filters using a Büchner funnel. The filtrates were subjected to SAFE distillation (water bath at 40 °C, apparatus body at 30 °C, pressure < 10^−3^ Pa). The distillate was continuously collected in a liquid nitrogen-cooled flask, recovered after approximately 25 min, and dried over anhydrous sodium sulfate. The extract was spiked with 25 μL of n-decane (50,000 µg·L^−1^) as an external standard (concentration in extract: 2500 µg·L^−1^) and concentrated to 500 μL using a Kuderna–Danish apparatus at 45 °C. Extracts were stored at −80 °C until GC–MS analysis.

#### 4.4.2. GC-MS Analysis and Quantification

SAFE extracts were analysed by gas chromatography–mass spectrometry (GC–MS). One microlitre of extract was injected in splitless mode into an Agilent 7890B gas chromatograph (Agilent Technologies, Santa Clara, CA, USA) coupled to a single-quadrupole mass spectrometer (Agilent 5977B). The injector temperature was set to 250 °C, with a splitless hold time of 0.8 min. Separation was achieved on a non-polar CP-Sil 5 CB column (50 m × 0.32 mm i.d., 1.2 μm film thickness; Agilent Technologies, Santa Clara, CA, USA). Helium (99.999% purity; Agilent carrier gas filter CP17973) was used as the carrier gas at a constant pressure of 100 kPa. The oven temperature program was as follows: from 36 to 85 °C at 20 °C·min^−1^, then to 145 °C at 1 °C·min^−1^, and finally to 250 °C at 3 °C·min^−1^, held for 30 min. The transfer line temperature was 250 °C. The mass spectrometer operated under electron impact ionisation (70 eV) in selected-ion monitoring (SIM) mode. The following equation was used for quantification, considering identical recovery factors between the analytes and the internal standard (IS): aroma concentration in sample (in μg·kg^−1^) = IS concentration in sample (in μg·kg^−1^) × (aroma area/IS area) × (IS response coefficient/amino acid response coefficient). The latter factor was determined by external calibration when analytical standards were available.

### 4.5. Statistical Analysis

Statistical analysis was performed with the JMP program (version 17), SAS Institute Inc., Cary, NC, USA. Sample analysis was performed in duplicate. Student tests were performed to determine significant differences between samples (*p* < 0.05).

## Figures and Tables

**Table 1:** Free amino acid levels (mg·kg^−1^) of roasted cocoa beans of six different origins (A: Madagascar, B: Brazil, C: Venezuela, D: Mexico, E: Cuba, F: Cameroon).

	Levels of Free Amino Acids (mg·kg^−1^)					
	Beans A	Beans B	Beans C	Beans D	Beans E	Beans F
Leucine/Isoleucine	5793 (±869)	4927 (±739)	3996 (±599)	4633 (±695)	4355 (±653)	2980 (±447)
Phenylalanine	2335 (±350)	2262 (±339)	1894 (±284)	2362 (±354)	1479 (±222)	1466 (±220)
Aspartic acid	1000 (±150)	1022 (±153)	756 (±113)	821 (±123)	742 (±111)	453 (±68)
Alanine	934 (±140)	1002 (±150)	781 (±117)	917 (±138)	1051(±158)	1010 (±152)
Tyrosine	888 (±133)	976 (±146)	794 (±119)	909 (±136)	721 (±108)	610 (±92)
Arginine	826 (±124)	777 (±117)	708 (±106)	508 (±76)	684 (±103)	667 (±100)
Glutamic acid	676 (±101)	601 (±90)	462 (±69)	500 (±75)	623 (±93)	548 (±82)
Valine	668 (±100)	635 (±95)	515 (±77)	581 (±87)	562 (±84)	846 (±127)
Threonine	664 (±100)	702 (±105)	523 (±78)	563 (±84)	511 (±77)	479 (±72)
Proline	429 (±64)	541 (±81)	458 (±69)	432 (±65)	440 (±66)	520 (±78)
Lysine	412 (±62)	373 (±56)	293 (±44)	208 (±31)	298 (±45)	315 (±47)
Histidine	146 (±22)	119 (±18)	79 (±12)	157 (±24)	80 (±12)	184 (±28)
Asparagine	80 (±12)	121 (±18)	136 (±20)	278 (±42)	62 (±9)	70 (±10)
Serine	62 (±9)	125 (±19)	96 (±14)	146 (±22)	77 (±12)	47 (±7)
Glycine	45 (±7)	174 (±26)	104 (±16)	133 (±20)	83 (±12)	237 (±36)
Tryptophan	44 (±7)	40 (±6)	29 (±4)	48 (±7)	31 (±5)	79 (±12)
Methionine	4 (±1)	46 (±7)	7 (±1)	71 (±11)	<LOQ	19 (±3)
Glutamine	<LOD	4 (±1)	<LOD	9 (±1)	3 (±1)	22 (±3)
**Total**	15,006 (±2250)	14,447 (±2167)	11,631 (±1744)	13,276 (±1991)	11,803 (±1170)	10,552 (±1582)

Standard deviation between duplicates was <15%. LOQ: limit of detection; LOD: limit of quantification.

**Table 2:** Levels of free amino acids (mg·kg^−1^) in the chocolate from beans A and the cocoa-free chocolate surrogate.

	Chocolate from Beans A	Cocoa-Free Chocolate Surrogate
Leucine/Isoleucine	8625 (±1294)	689 (±103)
Phenylalanine	4463 (±669)	297 (±45)
Alanine	2707 (±406)	110 (±17)
Valine	2356 (±353)	50 (±8)
Tyrosine	1749 (±262)	25 (±4)
Glutamic acid	1149 (±172)	115 (±17)
Threonine	1077 (±162)	12 (±2)
Aspartic acid	1039 (±156)	28 (±4)
Proline	890 (±134)	73 (±11)
Arginine	812 (±122)	17 (±3)
Glycine	491 (±74)	22 (±3)
Histidine	337 (±51)	22 (±3)
Tryptophan	307 (±46)	83 (±12)
Lysine	300 (±45)	10 (±2)
Asparagine	142 (±21)	8 (±1)
Serine	102 (±15)	2 (±0)
Glutamine	68 (±10)	7 (±1)
Methionine	13 (±2)	4 (±1)
**Total**	26,627 (±3994)	1571 (±236)

Standard deviation between duplicates was <15%.

**Table 3:** Origin and varieties of the analysed cocoa beans.

Letter	Origin	Variety for a Chocolate-Maker
A	Madagascar	*Criollo*/*Casse-Claire*
B	Brazil	*Forastero*
C	Venezuela	*Criollo*/*Chuao*
D	Mexico	*Criollo*/*Carmello*
E	Cuba	*Trinitario*
F	Cameroon	*Forastero*/*Amelonado*

The roasting conditions were as follows: 30 min at 140 °C.

## Data Availability

Data is contained within the article and Supplementary Materials.

## Supplementary Materials

The following supporting information can be downloaded at: https://www.mdpi.com/article/10.3390/molecules31071148/s1, Table S1. MRM transitions used to detect free amino acids by HPLC—MS/MS; Table S2. Amino acids groups and concentrations range for calibration.
