# Supplementary material for: Amino-Acid-Derived Toxins and Pyrazines in Chocolates and Cocoa-Free Chocolate Surrogates
Source: Molecules. 2026 Mar 31;31(7):1148. doi: 10.3390/molecules31071148 (PMC13074877; doi:10.3390/molecules31071148)
Supplement: Supplementary file 1 [file molecules-31-01148-s001.zip › molecules-4205592-supplementary.pdf]

molecules

Supplementary materials for: Amino-acid-derived toxins and pyrazines in chocolates and cocoa-free chocolate surrogates

Alexandre Dusart *, Lucie Villé, Thomas Vantsiotis, and Sonia Collin *

Unité de Brasserie et des Industries Alimentaires, Louvain Institute of Biomolecular Science and Technology (LIBST), Faculté des Bioingénieurs, Université catholique de Louvain, Louvain-la-Neuve 1348, Belgium
* Correspondence: alexandre.dusart@uclouvain.be; sonia.collin@uclouvain.be

Table S1. MRM transitions used to detect free amino acids by HPLC—MS/MS

Compound	Precursor ion (*m/z*)	Product ion (*m/z*)	Collision energy (V)	Retention time (min.)
Alanine	89.967	43.97[a]	11.3	0.947
Arginine	175.05	69.97[a]	16.96	2.825
		116.05	14.24	
Asparagine	132.967	73.97[a]	17.03	1.312
		87.13	9.87	
Aspartic acid	133.967	73.97[a]	14.95	1.131
		87.97	10.44	
Glutamic acid	147.967	83.97[a]	16.53	1.025
		130.05	9.87	
Glutamine	147.3	129.97[a]	9.87	1.273
		83.97	17.32	
Glycine	75.967	29.97[a]	11.59	1.262
Histidine	155.967	110.05[a]	14.17	2.784
		82.97	25.12	
Isoleucine	132.05	85.97[a]	10.8	0.405
Leucine	132.05	86.05[a]	10.51	0.405
Lysine	147.05	84.05[a]	16.89	2.843
		130.05	9.8	
Methionine	149.967	104.05[a]	10.94	0.508
Phenylalanine	165.967	119.97[a]	13.31	0.381
		103.05	28.06	
Proline	116.967	69.97[a]	16.03	0.693
		43.05	30.21	
Serine	105.967	59.97[a]	11.66	1.31
		42.13	22.9	
DL-3-Aminoisobutyric acid	103.967	86.05[a]	7.43	0.793
		30.05	15.74	

Valine	118.05	71.97[a]	11.37	0.579
		55.05	20.75	
Threonine	119.97	74.05[a]	11.16	1.063
		56.13	16.96	
Tryptophane	205.05	188.05[a]	10.01	0.456
		146.05	17.89	
Tyrosine	181.97	136.05[a]	13.45	0.637

[a]: *signal used for quantification*

Table S2. Amino acids groups and concentrations range for calibration

Group	Amino acids	Calibration range (mg·L^{-1})
High occurrence	Alanine, Arginine, Asparagine, Leucine, Isoleucine, Phenylalanine, Tyrosine, Valine	0.3; 0.9; 3; 6; 9; 12
Medium occurrence	Aspartic acid, Glutamic acid, Lysine, Proline, Serine, Threonine	0.3; 0.6; 1; 2; 4; 6
Low occurrence	Glycine, Glutamine, Histidine, Methionine, Tryptophan	0.1; 0.3; 0.5; 1; 2; 3
